# Toxicological parameters of albino rats fed with extruded snacks from Aerial yam (*Dioscoria bulbifera*) and African breadfruit seed (*Treculia africana*)

**DOI:** 10.1002/fsn3.533

**Published:** 2017-10-25

**Authors:** Kazeem K. Olatoye, Gibson L. Arueya

**Affiliations:** ^1^ Department of Food Science and Technology College of Agriculture Kwara State University Malete Nigeria; ^2^ Department of Food Technology Faculty of Technology University of Ibadan Ibadan Nigeria

**Keywords:** aerial yam, African breadfruit seeds, extruded snack, toxicology

## Abstract

In this study, safety of novel food from aerial yam and *Treculia africana*, underutilized food materials with high‐nutritive value and health benefits were investigated. Animal experiment involving the use of thirty (30) male albino rats was conducted for 28 days.Thereafter, rats in all groups were sacrificed and blood samples collected for biochemical analysis and hematological assay. Some vital organs were harvested and used for histological analysis. Biochemical and hematological parameters were not significantly *p *≤ .05 different among the treatment and controls. However there was an increase in monocytes, which is a reflection of immune boosting potential of the novel snack. No significant pathological changes were observed in liver and kidney of rats fed with this snack. Rats showed no signs of toxicity within the study period. These findings suggest that product may be safe and useful as an Immune adjuvant.

## 1 INTRODUCTION

The use of various underexploited food materials in product development is on the increase, probably due to growth in human population with resultant hike in prices of foods. Toxicological implications of such products and their phytoconstituents on consumers health are rarely investigated (Saad, Azaizeh, Abu Hijleh, & Said, 2006). Interactions between constituents in the food materials during processing and their adverse effect cannot be ruled out. Thus, it becomes imperative to ascertain the level of risk before launching such food product into market. One way of achieving this is through animal study. The use of small mammals such as rats whose body physiology and nutrient requirements closely resemble that of humans have been widely documented (Ani, Onweluzo, & Asogwa, 2012; Princewill‐Ogbonna, Abagha, & Ijioma, 2015). Toxicology of novel food from underutilized materials like aerial yam and African breadfruit seed can be examined this way. Aerial yam is a high yielding and nutritive, but underexploited yam species (Afiukwa & Igwe, 2015;; Polycarp, Afoakwa, Budu, & Otoo, 2012). It is also highly medicinal and was described as natural store of antioxidant against cancer (Oliver, 1989). It had been used to treat the diseases of the lungs, kidneys and spleen, and many types of diarrhea in Chinese system of medicine (Ahmed et al., 2009). It is traditionally used to lower glycemic index. It was also reported to provide a more sustained form of energy and protection against obesity and diabetes (Adewole, Ogunmodede, Adeniran, Talabi, & Lajide, 2011; Suriyavathana & Indupriya, 2011). Antihyperglycemic and antidyslipidemic activity of aqueous extract of its tubers has been documented (Ahmed et al., 2009). However, as with tubers generally it has a relatively low‐protein content (5.30–9.27%) and may require complementation with legumes, when used as food. African breadfruit seed is a cheap, nutritive and lesser‐known protein‐rich food material, (18–23%) which belongs to the family *Moraceae* (Appiah, Oduro, Ellis, & Adu, 2012). The essential amino acids composition especially for; lysine, leucine, threonine, and valine compare favorably with those of soybean (Nwabueze, 2007). Its fat content ranges between (9 and 12%) with unsaturated fatty acid comparable with those of melon‐seeds, soybeans, and groundnut oil (Ejiofor & Okafor, 1997; Ekpeyong, 1985; Olapade & Aworh, 2012). It is a relatively good source of iron, calcium, potassium, and riboflavin (ICRAF, 2010). Studies on its pharmacological properties revealed hypolipidaemic effects that reflected reduction in triglyceride, low density lipoprotein (LDL), and ability to boost High density lipoprotein (HDL) (Ogbonnia, Odimegwuand, & Enwuru, 2008; Osabor, Ogar, Okafor, & Egbung, 2009). It is also known for increased satiety and provides sense of fullness and encourages the individual to stop eating sooner, thereby reducing total energy intake.

Development of functional food from these two materials may alleviate the scourge of degenerative diseases such as overweight, obesity, and diabetes especially in the developing countries, where the cost of medication is quite unaffordable for many sufferers. However, knowledge of safety or otherwise of such product should be established as sparse information about this is available. The objective of this work was to evaluate the toxicology of extruded snack from aerial yam and African breadfruit seed.

## 2 MATERIALS AND METHODS

### 2.1 Sources of materials

Aerial yam was obtained from Genetic resource centre (GRC), International Institute of Tropical Agriculture (IITA), Ibadan, Oyo State, Nigeria. African breadfruit Seed was obtained from a commercial seller at Alaba international market, Lagos, Nigeria. Other ingredients like vanilla flavor, pepper, sucrose vegetable oil were purchase at Bodija Market, Ibadan.

### 2.2 Production of snack

**Figure 1:**
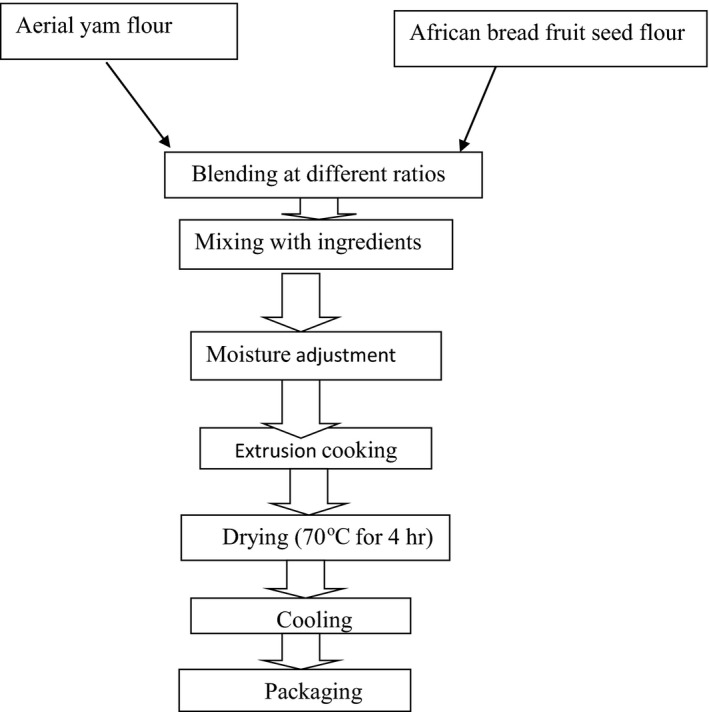
Flowchart for the production of extruded snacks from aerial yam and African breadfruit seeds

Aerial yam and African breadfruit were transformed into flour and combined at 100:00, 00:100, 80:20, and 65:35 levels, respectively. The blends were mixed with measured amount of additional ingredients as shown in Figure 1 The moisture was adjusted into 17.5% with material balance (Omeire, 2013) and the dough subjected to hot extrusion at barrel temperature (120°C) and the screw speed (70 rpm). The dried‐ cooled snacks were properly stored with polyethylene, pending the animal experiment.

### 2.3 Animals and treatment

Total number of thirty (30) male albino rats (*Rattus novergicus*) weighing between (100 and 200 g), obtained from the Animal House, Department of Clinical pharmacy, University of Ibadan were used. They were acclimatized for 10 days to well ventilated room at temperature 30 ± 4°C and relative humidity of 60%. They were housed in standard cages, fed *adlibitum* with standard rat feed (Ladokun Feeds Ltd., Ibadan, Nigeria) and clean water. All animal experiments were conducted in compliance with NIH guidelines for care and use of laboratory animal (pub. No. 58‐23, Revised 1985) as reported by Akah, Alemji, Salawu, Okoye, and Offiah (2009). The study was conducted at veterinary medicine Department, university of Ibadan.

### 2.4 Animal handling

Rats were randomly grouped into six groups (five animals each) and separately housed after adaptation period. Groups were provided with diets as shown in Table 2.1 and water offered to them *adlibitum* (Akande, Odunsi, Emiola, & Adedeji, 2014; Ani et al., 2012). All animals were inspected daily for appearance of signs of toxicity and possible deaths. The same level of hygiene was maintained throughout the 28 days experimental period. This duration was based on the clinical trial conducted by Ismail et al. (1998) in similar study. Thereafter, rats in all groups were sacrificed and blood samples collected by cardiac puncture into ethylene diamine tetracetic acid (EDTA) bottles for biochemical analysis and hematological assay. The animals were thereafter quickly dissected and the liver and kidneys harvested for histopathological assessment.

### 2.5 Experimental feed formulation

100% each of Aerial yam flour(AYF) and African bread fruit seeds flour (ABF) were used as bases for diets EE0 and EE1, respectively (Table 1). EE2 and EE3 were, respectively, formulated from (80:20) and (65:35) combinations of AYF and ABF. Diet EE4 was the normal (control) diet purchase from commercial producer (Ladokun feeds), while EE5 served as positive control to reflect the effect of casein substitution for ABF.

**Table 1:** Animal grouping and diets consumed

Group	EE0 (I)	EE1 (II)	EE2 (III)	EE3 (IV)	EE4 (V)	EE5 (VI)
AYF: ABF	100:00	00:100	80: 20	65:35	Normal diet	Casein

AYF, aerial yam flour; ABF, African breadfruit seeds flour.

### 2.6 Biochemical analysis

Some biochemical parameters (Total protein, Albumin, Globulin, Aspartate (AST), Alanine amino transferase (ALT), Alkaline phosphatate (ALP), urea and Creatinine) were determined on the whole blood, using Roche diagnostic kits.

### 2.7 Hematological analysis

Hematological (Mean Corpuscular Hemoglobin Concentate (MCHC), Neutrophil, Lympocytes, Monocytes, Eosinophils, Packed cell volume (PVC, Hemoglobin (Hb), (RBC),(WBC), Platelets) were evaluated by Sysmex Automated Hematology analyze KX‐21 (Sysmex Corporation, Kobe, Japan).

### 2.8 Histopathological analysis

After the induced bleeding, four rats from group (I–VI) were sacrificed through vascular dislocation and liver and Kidney was removed from the animals. They were weighed and subjected to Histopathological analysis (toxicity signs) after fixation in slides using the method of Adesiji (1999).

### 2.9 Statistical analysis of data

All tests were replicated and data obtained were statistically analyzed using a one‐way analysis of variance (ANOVA) and means were separated by Duncan's Multiple Range Test (DMRT) using the Statistical package for social science (SPSS) IBM VERSION 21.0 package. Significance was accepted at .05 probability level.

## 3 RESULTS AND DISCUSSIONS

### 3.1 Biochemical profile of rats fed with AYF/ABF based snacks

Investigated biochemical parameters in this study were not significantly *p* ≤ .05 different among the treatment and were consistent with those of controls (Table 2). Similarly, no significant difference exists between the Albumin and globulin ratios in all the groups and control. The changes in total protein, albumin, and globulin are indications of diminished synthetic function of the liver (Yakubu, Bilbis, Lawal, & Akanji, 2003). It might be a consequence of impaired hepatocellular function; low‐albumin content in the serum and may also suggest liver damage. Activities of bio‐indicator enzymes (ALP, AST, and ALT) in the kidney were not inhibited as can be observed from the fact that no significant *p* ≤ .05 difference exists between the treatment and control. This implies normal function of kidney and no evidence of probable renal impairment. Statistically, there were no significantly differences P ≤ 0.05 between the treatments and control in this study for both creatinine and urea. Creatinine is a break‐down product of creatine phosphate in muscle. It is usually produced at a fairly constant rate by the body and filtered out of the blood by the kidneys. If the filtering capacity of the kidney is deficient, creatinine blood levels rise (Guyton & Hall, 2001). Measurement of serum creatinine is the most commonly used indicator of renal function. However, significant elevation in the serum level of creatinine and urea may be a pointer to renal dysfunction (Oluwole, Oluwaseun, & Bukola, 2012). Urea is the major end product of protein catabolism in animals and is the primary vehicle for removal of toxic ammonia from the body. It is primarily produced in the liver and secreted by the kidneys. Urea determination is very useful for assessing kidney function and in general, increased urea levels are associated with nephritis, renal ischemia and urinary tract obstruction (Lynda et al., 2009).

**Table 2:** Biochemical profile of rats fed with AYF/ABF based snacks

Sample	Total protein	Albumin	Globulin	A/G	AST	ALT	ALP	Urea	Creatinin
EE0	6.70 ± 0.43^a^	2.13 ± 0.30^a^	4.58 ± 0.56^a^	0.47 ± 0.07^a^	43.75 ± 2.17^a^	32.75 ± 0.75^a^	115.5 ± 5.50^a^	14.20 ± 0.47^a^	0.68 ± 0.08^a^
EE1	7.05 ± 00.32^a^	1.90 ± 0.20^a^	4.63 ± 0.36^a^	0.41 ± 0.04^a^	43.00 ± 3.34^a^	30.75 ± 2.39^a^	121.00 ± 5.24^a^	13.73 ± 0.28^a^	0.58 ± 0.05^a^
EE2	7.50 ± 0.52^a^	2.58 ± 0.65^a^	4.93 ± 0.28^a^	0.53 ± 0.15^a^	43.25 ± 2.66^a^	30.75 ± 2.02^a^	115.75 ± 3.97^a^	16.63 ± 1.75^a^	0.80 ± 0.15^a^
EE3	6.65 ± 0.51^a^	2.20 ± 0.54^a^	4.68 ± 0.59^a^	0.49 ± 0.13^a^	41.50 ± 0.02^a^	31.50 ± 1.44^a^	116.25 ± 5.14^a^	14.80 ± 1.27^a^	0.75 ± 0.16^a^
EE4	7.00 ± 0.42^a^	2.43 ± 0.34^a^	4.58 ± 0.93^a^	0.58 ± 0.13^a^	40.75 ± 2.06^a^	29.75 ± 1.89^a^	107.75 ± 2.75^a^	15.90 ± 1.08^a^	0.85 ± 0.12^a^
EE5	7.13 ± 0.39^a^	2.60 ± 0.52^a^	4.53 ± 0.57^a^	0.60 ± 0.14^a^	36.75 ± 0.63^a^	27.50 ± 0.96^a^	109.75 ± 5.98^a^	16.33 ± 1.12^a^	0.80 ± 0.08^a^

Means with the same superscript in the same column are not significantly different ( *p *≤ .05).

### 3.2 Effect of AYF/ABF snacks consumption on some Red blood cell parameters

There were no significant *p* ≤ .05 differences between investigated red blood cell parameters (MCV, MCH, and MCHC) of rats fed with experimental diets compared with those of control (EE4) (Table 3). Slight variations were observed in PCV, HB and RBC, except for group fed with EE1 and EE2. However, all the red blood cell parameters were very close to expected normal range as reported by Akande et al.(2014).This finding showed that the snacks has no adverse effect on hematological parameters studied. These findings agree with that reported by Princewill‐Ogbonna et al., (2015) in study on rat fed with boiled and roasted aerial yam incorporated at varying proportions (20–40%) into commercial rat feed and the report of Akande et al. (2014), in similar study, titled “growth performance and hematological indices of rat fed with mucuna seeds meal‐based diet.” Extrusion cooking, like other heat treatment might have helped to ameliorate the negative effects of the antinutritional factors on these blood indices. Consumption of aerial yam and its substitution with African bread fruit seeds compared well with both the control and normal.

**Table 3:** Influence of AYF/ABF snacks consumption on some Red blood cell parameters

Samples	PCV (%)	HB (g/dl)	RBC × 10^12^/L	MCV (fl)	MCH (pg)	MCHC (g/L)
EE0	41.00 ± 0.71^bc^	13.50 ± 0.25^b^	6.77 ± 0.21^ab^	60.64 ± 0.89^a^	19.97 ± 0.29^a^	32.93 ± 0.15^a^
EE1	46.25 ± 1.03^a^	15.15 ± 0.29^a^	7.77 ± 0.24^a^	59.62 ± 0.69^a^	19.53 ± 0.25^a^	32.77 ± 0.15^a^
EE2	46.00 ± 2.20^a^	15.45 ± 0.29^a^	7.77 ± 0.45^a^	59.29 ± 0.69^a^	19.89 ± 0.21^a^	33.55 ± 0.37^a^
EE3	39.25 ± 2.02^c^	13.05 ± 0.65^b^	6.44 ± 0.42^b^	61.20 ± 1.70^a^	20.35 ± 0.54^a^	33.25 ± 0.13^a^
EE4	46.50 ± 1.19^a^	15.60 ± 0.38^a^	7.82 ± 0.30a	59.57 ± 0.78^a^	19.99 ± 0.37^a^	33.55 ± 0.22^a^
EE5	44.50 ± 1.25^ab^	14.55 ± 0.44^ab^	7.54 ± 0.23^a^	59.06 ± 1.00^a^	19.31 ± 0.27^a^	32.71 ± 0.64^a^
Normal	45.80 ± 2.7	14.30 ± 1.10	7.04 ± 0.81	65.40 ± 4.9	20.50 ± 1.3	31.30 ± 0.9

Means with the same superscript in the same column are not significantly different (*p* ≤ 0.05).

### 3.3 Influence of AYF/ABF snacks consumption on some white blood cell parameters

There were no significant *p* ≤ .05 differences between white blood cell parameters and the platelets of rats fed with experimental diet and those fed with control diet, except monocytes (Table 4). It was observed that monocytes of group fed with 80:20(EE2) and 65:35(EE3) blends of aerial yam and African breadfruit seeds flour were 2.50% and 2.00%, which are higher than that of control and casein groups. Rat fed with 100% AYF also has higher monocytes (2.00%) than the control groups (1.75%). This is a positive immunomodulatory potential. Moreover, the ratios of AST to platelets were significantly different *p* ≤ .05, with control being the least and EE2 the highest. Similar results were obtained by (Princewill‐Ogbonna et al., 2015). This result reflects the potential health benefits of the snacks to the consumer. These benefits may be attributed to presence of bioactive phytochemicals such as phenols, which are known to possess functional properties such as anticarcinogenic, antiviral, antimicrobial, anti‐inflammatory, hypotensive, and antioxidant activity (Shajeela, Mohan, Jesudas, & Sori, 2011; Shetty, 1997).

**Table 4:** Influence of AYF/ABF snacks consumption on some white blood cell parameters

Samples	WBC × 10^3µl^	Lymphocyt %	Neutrophils %	Monocytes %	Eosinophils%	Platelets	AST/Platelet
EE0	6.81 ± 1.09^a^	61.00 ± 3.16^a^	34.75 ± 3.22^a^	2.00 ± 0.41^ab^	2.25 ± 0.25^a^	1.33 ± 0.13^a^	0.34 ± 0.03^bc^
EE1	7.55 ± 0.52^a^	60.25 ± 2.32^a^	36.25 ± 2.02^a^	1.50 ± 0.38^b^	2.00 ± 0.58^a^	1.35 ± 0.10^a^	0.32 ± 0.03^bc^
EE2	6.16 ± 1.52^a^	67.50 ± 3.97^a^	28.50 ± 4.44^a^	2.75 ± 0.25a	1.25 ± 0.96^a^	0.70 ± 0.20^a^	0.55 ± 0.03^a^
EE3	5.43 ± 1.04^a^	57.50 ± 3.78^a^	28.50 ± 4.09^a^	2.00 ± 0.41^ab^	1.15 ± 0.58^a^	0.99 ± 0.12^a^	0.45 ± 0.04^ab^
EE4	8.23 ± 0.93^a^	66.00 ± 3.42^a^	30.50 ± 3.43^a^	1.75 ± 0.25^ab^	1.41 ± 0.71^a^	1.55 ± 0.13^a^	0.27 ± 0.03^c^
EE5	6.74 ± 2.27^a^	62.74 ± 2.74^a^	34.50 ± 3.30^a^	1.75 ± 0.49^ab^	1.75 ± 0.25^a^	0.89 ± 0.29^a^	0.40 ± 0.06^bc^

Means with the same superscript in the same column are not significantly different (*p *≤ 0.05).

### 3.4 Liver and kidney histology of rats fed with varying ratio of AYF/ABF based snack

**Figure 2:**
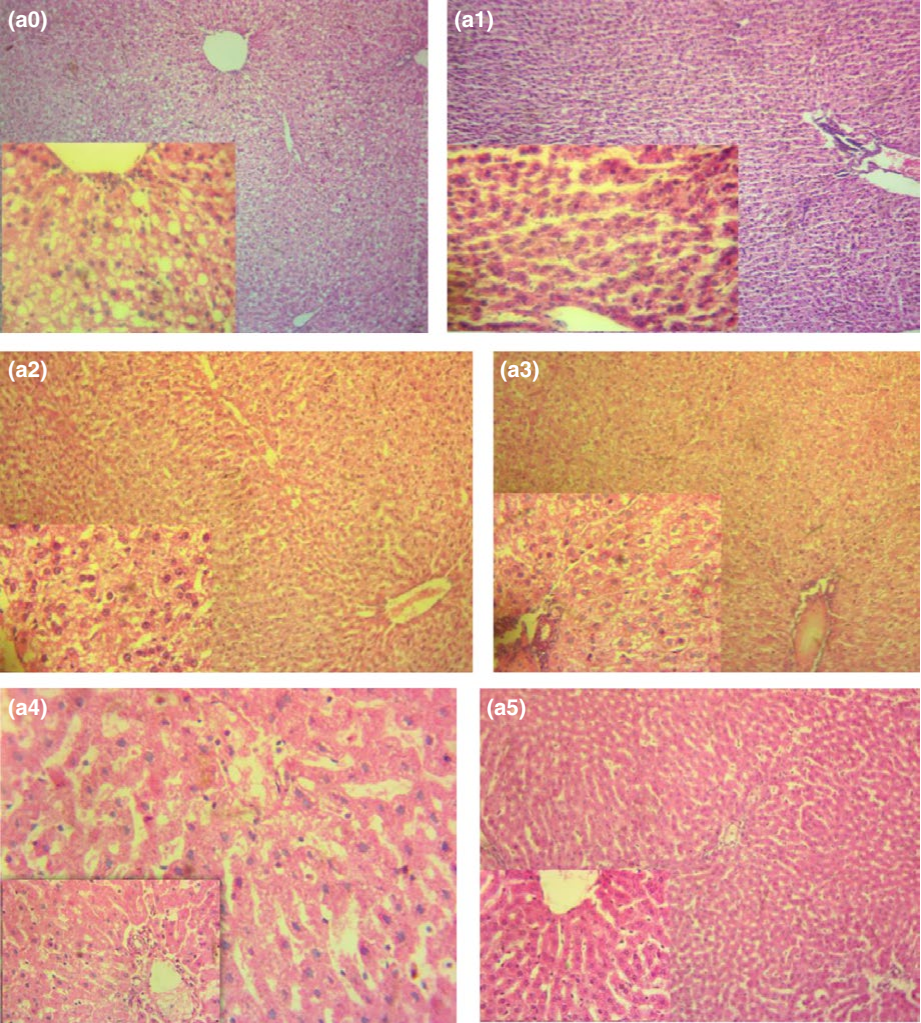
Liver Histology of rats fed with AYF‐ABF based Snack:(a0)Photomicrograph of the liver of rat with 100%AYF with no traces of necrosis and lesions; (a1)Photomicrograph of the liver of rats fed with100% ABF showing no evidence of necrosis and lesions; (a2)Photomicrograph of the liver of rats fed with 80%AYF and 20% ABF also showing no necrosis and lesions; (a3)Photomicrograph of the liver of rat fed with 65%AYF and 35%ABFwithnormal appearance; (a4)Photomicrograph of the liver of rat fed with normal diet showing normal appearance; (a5)Photomicrograph of the liver of rat fed with AYF and Casein showing normal appearance. Key: Mag x100, Inset: X400

**Figure 3:**
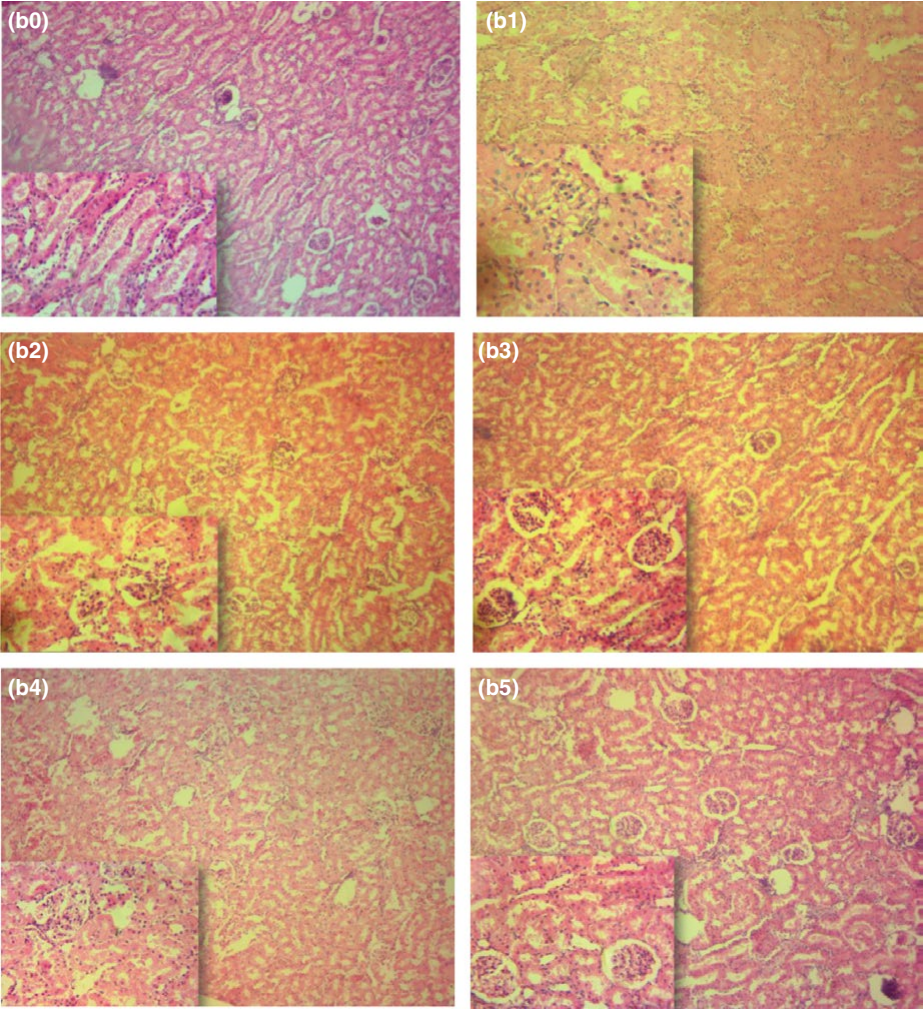
Kidney Histology of rats fed with AYF‐ABF based Snack: (b0)Photomicrograph of the kidney of rat fed with100%AYF showing no form of necrosis and interlobular oedema; (b1)Photomicrograph of the kidney of rats fed with 100% ABF, showing no necrosis and lesions; (b2)Photomicrograph of the kidney of rats fed with 80%AYF and 20%ABF showing no necrosis and lesions; (b3) Photomicrograph of the kidney of rat fed with 65%AYF and 35%ABFwith normal appearance;(b4)Photomicrograph of the kidneyof rat fedwith normal diet showingnormal appearance; (b5)Photomicrograph of the kidney of rat fed with AYF and casein showing normal appearance. Key: Mag x100, Inset x400

There were no significant pathological changes in liver and kidney of rats fed AYF/ABF based snacks, especially at 65:35 combinations of Aerial yam and African breadfruit seeds. The hepatocytes in the liver showed no form of necrosis or lesions and any histopathological alterations and distortions (Figure 2). Similar trend was observed for the kidney histology. There was no sign of glomerular shrinking nor hemorrhage, necrosis, glomerular cells shrinking, and interstitial pneumonia (Figure 3). Findings of this study revealed that rats fed with snacks produced from Aerial yam, African breadfruit seeds and their blends showed no sign of toxicity within the 28 days of treatment period. Instead, they appeared physically, mechanically, and emotionally normal and healthy.

### 3.5 Conclusions and recommendations

Biochemical (Total protein, Albumin, Globulin, Aspartate (AST), Alanine amino transferase (ALT), Alkalinephosphatate (ALP)urea and Creatinine), Heamatological (Mean Corpuscular Hemoglobin Concentate (MCHC) Neutrophil, Lympocytes, Monocytes, Eosinophils, Packed cell volume (PVC, Hemoglobin (Hb), (RBC), (WBC), Platelets), and Histological (Liver and Kidney) parameters of the animals were within normal range and compared very well with those in control groups, with no signs of toxicity, thereby suggesting the safety of the snack in animals. It can therefore be safely recommended for clinical trials in human.

## CONFLICT OF INTEREST

No conflict of interest is hereby declared by the authors.
